# Solution-Processed Donor-Acceptor Polymer Nanowire Network Semiconductors For High-Performance Field-Effect Transistors

**DOI:** 10.1038/srep24476

**Published:** 2016-04-19

**Authors:** Yanlian Lei, Ping Deng, Jun Li, Ming Lin, Furong Zhu, Tsz-Wai Ng, Chun-Sing Lee, Beng S. Ong

**Affiliations:** 1 Department of Physics and Institute of Advanced Materials, Hong Kong Baptist University, Hong Kong SAR, P. R. China; 2 Research Centre of Excellence, Institute of Creativity and Department of Chemistry, Hong Kong Baptist University, Hong Kong SAR, P. R. China; 3 Institute of Materials Research and Engineering, Agency for Science, Technology and Research, Singapore 117602; 4 Center of Super-Diamond and Advanced Films, Department of Physics and Materials Science, City University of Hong Kong, Hong Kong SAR, P. R. China; 5 Department of Materials Science and Engineering, South University of Science and Technology of China, Shenzhen, Guangdong, P.R. China

## Abstract

Organic field-effect transistors (OFETs) represent a low-cost transistor technology for creating next-generation large-area, flexible and ultra-low-cost electronics. Conjugated electron donor-acceptor (D-A) polymers have surfaced as ideal channel semiconductor candidates for OFETs. However, high-molecular weight (MW) D-A polymer semiconductors, which offer high field-effect mobility, generally suffer from processing complications due to limited solubility. Conversely, the readily soluble, low-MW D-A polymers give low mobility. We report herein a facile solution process which transformed a lower-MW, low-mobility diketopyrrolopyrrole-dithienylthieno[3,2-b]thiophene (I) into a high crystalline order and high-mobility semiconductor for OFETs applications. The process involved solution fabrication of a channel semiconductor film from a lower-MW (I) and polystyrene blends. With the help of cooperative shifting motion of polystyrene chain segments, (I) readily self-assembled and crystallized out in the polystyrene matrix as an interpenetrating, nanowire semiconductor network, providing significantly enhanced mobility (over 8 cm^2^V^−1^s^−1^), on/off ratio (10^7^), and other desirable field-effect properties that meet impactful OFET application requirements.

Fabrication of organic field-effect transistor (OFET) arrays and circuitries for use in next-generation large-area, flexible and ultra-low-cost electronics necessitates a readily solution-processable semiconductor with functionally capable performance characteristics^1^,^2^,^3^,^4^,^5^,^6^,^7^. This is because solution processability potentially enable low-cost manufacturing of electronic devices through high-throughput roll-to-roll printing processes. In this regard, organic conjugated polymers have been perceived to be ideal semiconductor candidates for OFETs as they appear to possess desirable transistor functionality and solution processability and mechanical and film-forming properties suitable for roll-to-roll printing^4^,^5^. Over the past several years, great strides have been made in polymer semiconductor design and fabrication processes, expediting progress towards realization of low-cost OFETs^8^,^9^,^10^,^11^,^12^,^13^,^14^,^15^,^16^,^17^. The enhancement in field-effect mobility of OFETs has been particularly phenomenal, from well below 1 cm^2^ V^−1^s^−1^ just a few years ago to over 10 cm^2^ V^−1^s^−1^ and beyond today^9^,^10^,^11^,^12^,^13^,^14^,^15^,^16^,^17^,^18^,^19^,^20^,^21^,^22^,^23^,^24^. In addition, significant improvements in other transistor properties such as device’s ambient and operational stability, etc. have also been demonstrated^10^,^14^.

However, there remain several critical materials and process challenges yet to be resolved for practical applications. From the fabrication perspective, the often tedious or specialized semiconductor processing to promote higher crystallinity and long-range ordering for high charge carrier mobility would be costly, if not impossible to scale^18^,^19^,^20^. This would negate the fundamental economic benefits of OFETs. From the materials perspective, it is known that the charge carrier mobility of polymer semiconductors increases with molecular weights (MW), and attainment of high mobility requires high-MW materials^10^,^11^. Unfortunately, high-MW polymer semiconductors generally possess limited solubility in common processing solvents^10^. Complications such as solution gelling, solid precipitation, etc. often arise during semiconductor fabrication leading to non-uniform channel semiconductor film formation. Consequently a wide variability in OFET performance results when a high-MW polymer semiconductor is utilized. Another practical difficulty is the synthetic challenge in obtaining high-MW polymer semiconductors due to premature precipitation of lower-MW polymer intermediates out of reaction media during synthesis. It would thus be highly desirable and of great practical significance if a soluble, low-MW polymer semiconductor can be processed into a channel semiconductor of higher crystalline orders to improve its mobility. This would resolve both the process and materials difficulties, thus facilitating OFET technology’s transition from laboratory to marketplace.

Facilitated crystal growth of organic semiconductors to enhance their charge transport efficacy have been reported^22^,^23^,^24^,^25^,^26^,^27^,^28^,^29^,^30^,^31^,^32^,^33^. These are primarily solvency-controlled crystallization from “marginal” solvents or phase separation of semiconductor molecules into higher crystallinity in inert polymer blends^20^,^29^,^32^. However, many of these reported processing procedures to enhance charge transport properties of organic semiconductors are neither amenable to common printing techniques nor readily scalable for practical adoption. In this work, we report our studies on medium-assisted self-assembly of a solution processable, lower-MW, low-mobility diketopyrrolopyrrole-dithienylthieno[3,2-b]thiophene (DPP-DTT) polymer semiconductor, (I) as shown in Fig. 1, into high crystalline orders in a semiconductor film system for enhanced charge transport efficacy. The saturated field-effect mobility of lower-MW (I) was as low as 10^−3^ cm^2^ V^−1^s^−1^ 21, while those of high-MW materials could be high as over 10 cm^2^ V^−1^s^−1^ 10. However, the high-MW polymer has severe processing difficulties, thus rendering its practical application highly questionable. Through our studies, we have succeeded in creating a long-ranged nanowire semiconductor network of high crystalline orders from a lower-MW (I) through a facile solution process. This has dramatically propelled the field-effect mobility and on/off ratio of lower-MW (I) from about 1.5 cm^2^ V^−1^s^−1^ and 10^5^ respectively to over 8 cm^2^ V^−1^s^−1^ and 10^7^ respectively under ambient conditions. These OFET properties are functionally more than sufficient for many impactful electronic applications (e.g., display backplane electronics, ultra-low-cost radio-frequency identification tags, etc.)^17^.

## Results and Discussion

Lower-MW (I) was soluble in common organic solvents with heat, particularly in chlorinated solvents such as chloroform and chlorobenzene. The present process involved dissolution of a lower-MW (I) (~199 kg mol^−1^) and polystyrene (2.2 kg mol^−1^) in dichlorobenzene at a polystyrene loading of from 20 to 80 weight percent (wt%). The resulting solution was spin cast on octadecyltrichlorosilane (OTS-18)-modified silicon wafer (SiO_2_/Si) substrates to form a polymer thin film, air-dried, and optionally thermally annealed at 200 °C for 5 min. Unlike earlier reported vertical phase separation of semiconductor/polymer blends which separated into two layers^25^,^28^,^29^,^30^,^31^,^33^, (I) in the polystyrene blend underwent segregation and self-assembly into higher crystalline orders and crystallized out within the polystyrene matrix. The polystyrene in the blend helped promote the molecular self-assembly and crystallization of (I), and the achievement of long-range, high crystalline orders was greatly facilitated through thermal treatment above the glass transition temperature (Tg) of polystyrene. This was fundamentally different from and superior to the attainment of higher crystalline orders of polymer semiconductors in a marginal solvent^20^ or with the aid of a high-boiling solvent in the coating solution^27^. In these cases, most of the solvents evaporated off during spin-coating and drying, while the polystyrene in the present case remained in the system and permitted further molecular organization of (I) during subsequent thermal annealing, leading to much higher crystallinity and longer-range orders.

### Spectroscopic and microscopic characterizations

**Figure 1:**
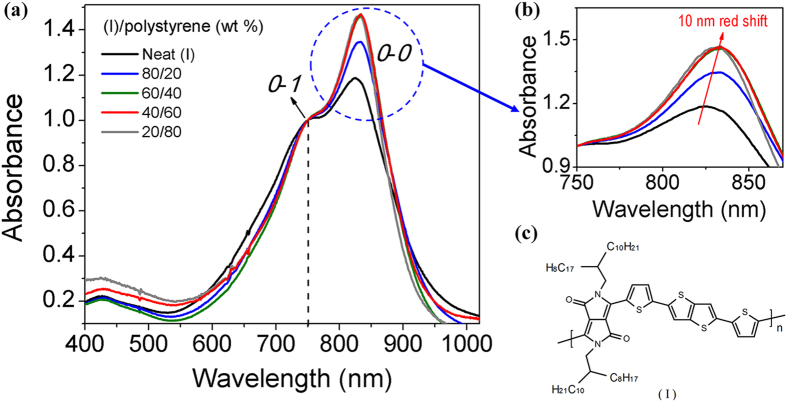
Thin-film UV-vis adsorption spectra of (I) and its blends with polystyrene. (**a**) Increase in absorbance of absorption peaks at ~825 nm in polystyrene blended films (spectra normalized at ~750 nm); (**b**) red-shifts of ~825 nm-absorption maxima of polystyrene blended films from that of neat (I) film; and (**c**) Chemical structure of DPP-DTT, (I).

The high molecular orderings of (I) in the polystyrene blended films were reflected spectroscopically in their absorption spectral behaviors. UV-visible absorption spectra of (I)/polystyrene films displayed the usual dual-band absorption of electron donor-acceptor (D-A) polymer semiconductors with the charge transfer band absorption at ~740–835 nm showing vibronic splitting characteristic of highly ordered structures^15^,^34^,^35^,^36^ (Fig. 1a). With increasing polystyrene loading, the 0-0 vibrational peak (~825 nm) exhibited progressive increase in intensity at the expense of 0–1 vibrational peak intensity (~750 nm), and the increase was most pronounced at about 60 wt% polystyrene. This increase in 825-nm intensity was concomitant a slight spectral red-shift of about 10 nm (Fig. 1b). These were obviously the manifestations of higher molecular orderings from formation of lamellar stacks of (I)^35^ in the polystyrene blended films. The red-shift was similar to those observed in other semiconductor polymeric systems, which were attributed to intermolecular π–π stacking^15^,^27^.

**Figure 2:**
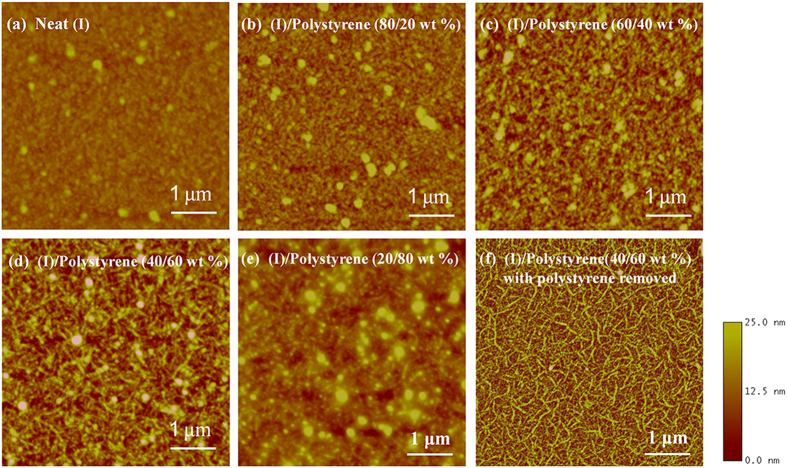
AFM topographic images of thermally annealed films of (I) with and without polystyrene. (**a**) Film of neat (I) displaying grainy nanodomain features; (**b**) (I)/polystyrene (80/20 wt%) film showing signs of aggregation of nanograins into nanowire-like structures; (**c**) formation of nanowire network in (I)/polystyrene (60/40 wt%) film; (**d**) optimal nanowire network formation in (I)/polystyrene (40/60 wt%) film; (**e**) degradation of nanowire domains in (I)/polystyrene (20/80 wt%) film; (**f**) well-defined nanowire network of thermally annealed (I)/polystyrene (40/60 wt%) film with the polystyrene removed.

To understand in greater detail the medium-assisted structural ordering and crystallization of (I), atomic force microscopic (AFM) studies of the semiconductor films were conducted. In AFM topographic images, the neat (I) film displayed mostly grainy, defused nanodomain features without any long-range orders (Fig. 2a). On the other hand, the polystyrene blended films showed progressive formation of larger, more defined, nanowire-like structures composed of aggregates of nanodomain features with diameter of 50–60 nm (Fig. 2b,c). Distinctive intertwined, long-ranged nanowire network structure with nanowire lengths of over 500 nm was observed at around 60 wt% polystyrene loading (Fig. 2d). Beyond 60 wt% polystyrene loading, degradation in crystalline domain structures occurred leading to decreased nanowire density (Fig. 2e).

The critical role of polystyrene in the self-assembly and structural ordering of (I) was evident when (I) was thermally annealed with or without polystyrene. To visualize the structural detail of (I) in (I)/polystyrene films (as cast and thermally annealed), the (I)/polystyrene film was first soaked in toluene for 2 minutes to dissolve and extract out the polystyrene component. The resulting “film” with the polystyrene essentially removed was then withdrawn from toluene and dried with nitrogen gas. Figure S1a,b (Supplementary Information) are the AFM and scanning electron microscopy (SEM) images of (I) from (I)/polystyrene (40/60 wt%) film thermally annealed (200 °C for 5 min.) after polystyrene removal (i.e., annealed in the absence of polystyrene), showing defused nanowire domain structures. In contrast, well-defined nanowire network features of (I) with nanowire lengths as long as over 1 μm could clearly be visualized (Fig. 2f, Figure S1d) when polystyrene was removed from thermally annealed (I)/polystyrene (40/60 wt%) film (i.e. annealed in the presence of polystyrene). These results strongly suggested that in the presence of polystyrene at temperatures above Tg of polystyrene, the cooperative shifting motion of polystyrene chain segments^37^ helped propel the movement of polymer chains of (I), facilitating their self-assembly into nanowire network structures. In essence, the polystyrene behaved like a fluid medium, permitting crystallization of (I) to propagate to the extent when highly ordered, crystalline structures “precipitated” out in the polystyrene matrix.

**Figure 3:**
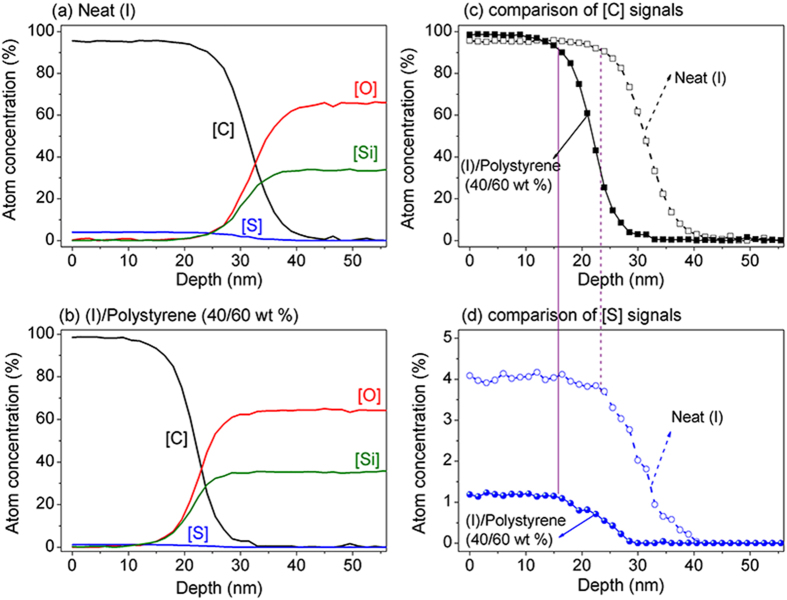
X-ray photoelectron spectroscopy (XPS) depth profiles of neat (I) and (I)/polystyrene (40/60 wt%) films. (**a**,**b**) are depth profiles of elements (C, S, O and Si) in respectively neat (I) and (I)/polystyrene film; (**c**) relative concentrations of carbon as a function of film depth in neat (I) and (I)/polystyrene film; and (**d**) relative concentrations of sulfur as a function of film depth in neat (I) and (I)/polystyrene film.

X-ray photoelectron spectroscopy (XPS) depth profiling analysis revealed a relatively uniform distribution of (I) in the (I)/polystyrene blend film. Figure 3a,b showed the relative ratios of carbon, sulfur, oxygen and silicon elements as a function of the film depth for neat (I) and (I)/polystyrene (40/60 wt%) films. The carbon signals came from both (I) and polystyrene, while the sulfur signal was from (I). The oxygen and silicon signals were primarily from the silicon wafer substrate with a very minor contribution from (I). In both cases, the carbon signals were present across the film thicknesses and decreased gradually and disappeared towards the bottom of the films (Fig. 3c), indicating that both polystyrene and (I) were present throughout the bulk of the films. Likewise, the blown-up figure (Fig. 3d) displayed relatively uniform distribution of sulfur signal across the thickness of the film, affirming relatively uniform distribution of nanowire network of (I) in the (I)/polystyrene blend film. This was distinctively different from vertical phase separation where a dual-layer film structure resulted^28^,^29^,^30^,^31^. In particular, vertical phase separation of a polymer semiconductor from the polymer blend did not afford higher structural orders of the semiconductor^31^.

**Figure 4:**
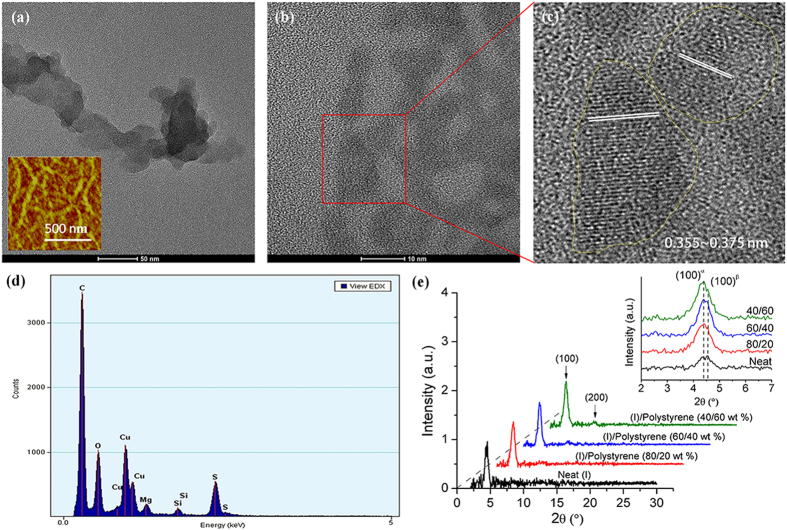
Transmission Electron Microscopy (TEM) images and EDX analysis of (I) from a thermally annealed (I)/polystyrene (40/60 wt%) film after polystyrene removal and Grazing Incidence X-ray Diffraction (GIXRD) of (I) and its polystyrene blends. (**a**) TEM image of a nanowire fragment of (I) showing aggregate of nanodomain structures; insert displaying AFM topographic image of portion of corresponding nanowire network; (**b**) crystalline domains within nanodomain structure of (I); (**c**) visually distinctive π-π stacking structures of the crystalline domains of (I); (**d**) EDX analysis showing presence of sulfur element in the nanowire network of (I); and (**e**) GIXRD diffraction patterns of (I) as a function of polystyrene loading, showing appearance of (200) diffraction as polystyrene loading increased; insert: increase in (100)^α^ phase intensity and concomitant decrease in (100)^β^ phase intensity with increased polystyrene loading.

Transmission electron microscopic (TEM) analysis of nanowire network of (I) was carried out to gain an insight into the nanowire structure of (I). The thermally annealed (I)/polystyrene (40/60 wt%) film on OTS-18-modified silicon wafer substrate was first soaked in toluene for 2 minutes to remove the polystyrene. The remaining nanowire network film of (I) was carefully removed and placed on a lacey carbon coated Cu grid and subject to TEM examination. Figure 4a showed a low-magnification TEM image of a segment of a nanowire composed of an aggregate of irregular nanodomain structures of 30–80 nm in size, and a portion of the corresponding intertwined nanowire network of (I) could be seen in the AFM topographic image provided in Fig. 4a insert. Energy-dispersive X-ray (EDX) spectroscopy (Fig. 4d) of the film revealed presence of sulfur element in these nanodomain structures, confirming the identity of (I) for the nanowire network^10^. A high-definition TEM image of one of nanodomain structures showed highly crystalline domains within the structure (Fig. 4b) with visually discernable π-π stacking features having a π-π distance ranging from about 0.355–0.375 nm (Fig. 4c). No particular preferential orientations in the plane normal to the π-π stacking were adopted by these π-π stacking domains. Accordingly, this polycrystalline nanowire structure was quite different from the single-crystal nanowires where polymer chains were arranged along the length of the wire with preferential π-π stacking direction normal to the length of the nanowire^20^.

### X-ray diffraction measurements

Out-of-plane grazing incidence X-ray diffraction (GIXRD) measurements (Fig. 4e) were conducted to further explore the structural transitions of (I) in the presence of polystyrene. The neat (I) film showed a weak and broad (100) diffraction with two peaks corresponding to two polymorphic phases, namely (100)^α^ (α-phase) at 2θ = 4.34° and (100)^β^ (β-phase) at 2θ = 4.55° (Fig. 4e, insert). The presence of polymorphic phases, observed previously in other copolymer systems^38^,^39^, was thought to be damaging to charge transport due to the charge-carrier scattering or blocking effects in the channel region. As the polystyrene loading increased, an increase in α-phase intensity with a concomitant decrease in β-phase intensity was observed. At 60 wt% polystyrene loading, the (100) diffraction became predominantly α-phase diffraction. In addition, (200) diffraction at 2θ = 8.68° also became discernable with increased polystyrene loadings, indicating increased crystallinity of (I) in the (I)/polystyrene film (Fig. 4e). These results were consistent with the formation of long-range nanowire network (Fig. 2d,f) in the (I)/polystyrene (40/60 wt%) film, which would be conducive to charge carrier transport. No π-π stacking diffraction at 2θ ~ 25° was noted in the out-of-plane XRD pattern of the film (Fig. 4e), reflecting that (I) assumed an edge-on orientation relative to the substrate even within the nanowire network.

### OFET performance characteristics

**Figure 5:**
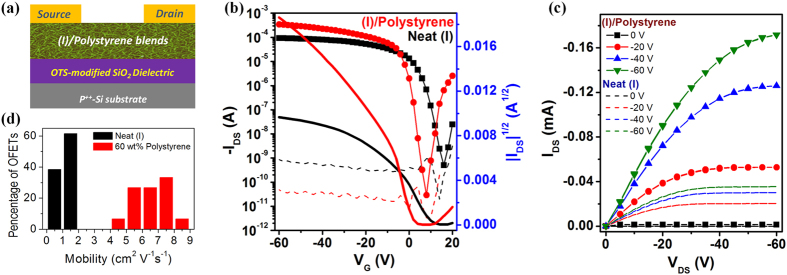
Field-effect transistor properties of OFETs with (I) and (I)/polystyrene semiconductor films. (**a**) Bottom gate-top contact experimental transistor structure; (**b**) typical transfer characteristics and (**c**) output curves of OFETs with (I) and (I)/polystyrene (40/60 wt%) channel semiconductors; (**d**) mobility distributions of OFETs with (I) and (I)/polystyrene (40/60 wt%) channel semiconductors.

The electrical performance characteristics of the (I)/polystyrene films as channel semiconductors in OFETs were investigated using a bottom gate-top contact transistor configuration (Fig. 5a). The transfer and output curves of OFETs with (I) and (I)/polystyrene (40/60 wt%) channel semiconductor films are shown in Fig. 5b,c, and the extracted field-effect mobility in the saturated regime, on/off ratio, threshold voltages summarized in Table 1. For completeness, typical transfer and output curves of OFETs with (I)/polystyrene films of various polystyrene loadings as channel layers are also given in Figure S2 (Supplementary Information). As noted, the mobility and on/off ratio increased with polystyrene loading in the (I)/polystyrene film up to about 60 wt%, where a mobility as high as 8.25 cm^2^ V^−1^s^−1^ and on/off ratio of ~10^7^ were attained. These results represented more than a factor of five higher in mobility and two orders of magnitude better in on/off ratio than similar devices with neat (I) as channel semiconductor (mobility ~1.5 cm^2^ V^−1^s^−1^ and on/off ratio ~10^5^). Furthermore, the average mobility (average ~6.76 cm^2^ V^−1^s^−1^) was comparable to those of high-MW (~500 kg mol^−1^) materials even though the π-π stacking distance was somewhat larger^10^. The substantially higher mobility correlate very well with the beneficial, predominantly single α-polymorph phase and interpenetrating nanowire network of (I) in polystyrene matrix, which served as a long-range percolated pathway for efficient charge transport between source/drain electrodes. The extremely high on/off ratio was attributable to the insulating nature of the polystyrene matrix which helped suppress leakage current (Fig. 5b). We also noted that the threshold voltage (V_th_) shift decreased with increased polystyrene loading in the (I)/polystyrene film (Table 1), and smallest positive threshold shift of 1.7 V was observed at 60 wt% polystyrene loading. In addition, the (I)/polystyrene devices exhibited smaller hysteresis effect than those with neat (I) semiconductor (Fig. S2a–e), again with the 60 wt% polystyrene devices displaying the smallest hysteresis effect. These results further demonstrate the definitive role of polystyrene in helping eliminate charge trapping at the polymer/SiO_2_ interface by passivating the surface of OTS-18-modified SiO_2_ dielectric. Beyond 60 wt% polystyrene loadings, degradation in charge transport properties was observed. This was largely due to the dilution effect, disrupting the interconnectivity of the nanowire network as could be seen in the (I)/polystyrene (20/80 wt%) film where the nanowire network appeared to be significantly degraded (Fig. 2e). The observed mobility was much lower at 3.34 cm^2^ V^−1^s^−1^ while maintaining the same high on/off ratio of ~10^7^ and relative small positive threshold shift of 4.6 V owing to the insulating effect of polystyrene.

It would be of interest to note that the nanowire network of (I) obtained after removing polystyrene from thermally annealed (I)/polystyrene (40/60 wt%) film (Fig. 2f or Figure S1c) gave a high mobility of 7.65 cm^−2^ V^−1^s^−1^ (Figure S3a in supplementary Information). This was comparable to those of OFETs based on a thermally annealed (I)/polystyrene (40/60 wt%) film (Fig. 2d). In contrast, OFETs with nanorod-like semiconductors from (I)/polystyrene (40/60 wt%) film thermally annealed subsequent to polystyrene removal (Figure S1a) yielded somewhat lower mobility of 4.36 cm^−2^ V^−1^s^−1^ (Figure S3b in supplementary Information), which was still much higher than that of OFETs with neat (I) as channel semiconductor. These results unequivocally affirm the critical role of polystyrene in facilitating achievement of long-range, higher crystalline orders of (I) in the (I)/polystyrene system for efficient charge transport efficacy.

### Effects of polystyrene MW on FET mobility

**Figure 6:**
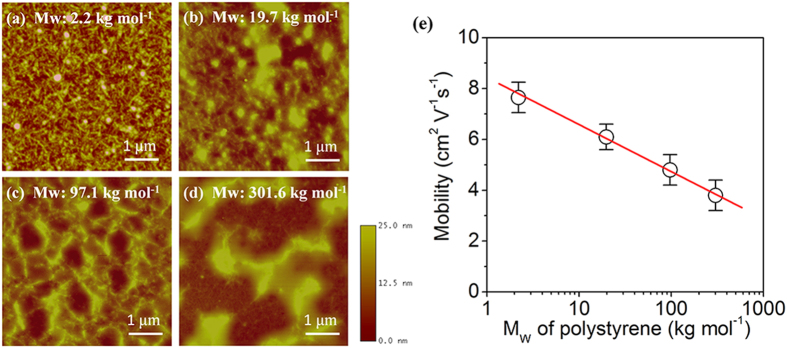
AFM topographic images of thermally annealed films of (I) in monodispersed polystyrenes of various MWs. (**a**) Mw of polystyrene = 2.2 kg mol^−1^; (**b**) Mw of polystyrene = 19.7 kg mol^−1^; (**c**) Mw of polystyrene = 97.1 kg mol^−1^; and (**d**) Mw of polystyrene = 301.6 kg mol^−1^; (**e**) Field-effect mobility as a function of Mw of polystyrene, showing inverse exponential dependence of mobility of (I) on Mw of polystyrene.

Further support for polystyrene as a fluid medium for crystallization of (I) came from the dependence of FET mobility of (I)/polystyrene semiconductor on polystyrene MW. The molecular orders of (I) in (I)/polystyrene film and thus the mobility would be expected to decrease as the viscosity of the medium or MW of polystyrene increase since higher viscosity would hamper the movement of (I), thus inhibiting its self-assembly into higher crystalline orders. For our studies, we used monodispersed polystyrenes of Mw ranging from 2.2 to 301.6 kg mol^−1^ at a polystyrene loading of 60 wt%. AFM topographic images of thermally annealed films of (I)/polystyrene showed presence of nanowire network features in the film with polystyrene Mw of 2.2 kg mol^−1^ (Fig. 6a). The network features of (I) degraded gradually as the Mw of polystyrene went up, and became non-existent beyond Mw of ~20 kg mol^−1^, when isolated islands or blocks of (I) appeared within the polystyrene matrix (Fig. 6b–d). These observations were consistent with the impediment of self-assembly of (I) into higher crystalline orders as the viscosity or Mw of polystyrene was increased. Consequently, degradation in field-effect mobility with increasing Mw of polystyrene was also observed (Fig. 6e), suggesting that low-viscosity or low-Mw polystyrene matrix would be most efficient in facilitating achievement of long-range crystalline orders of (I). With the ~300 kg mol^−1^-polystyrene in the (I)/polystyrene semiconductor composition, the mobility was still much higher than that of neat (I), reflecting the benefits of polystyrene incorporation. It was observed that while phase separation of nanowire domains occurred with high polystyrene MWs (Fig. 6c,d), extensive nanowire domain overlaps or connectivity existed. This may explain for the observed high FET mobility of the (I)/polystyrene semiconductors as compared to neat (I) semiconductor.

## Conclusions

In summary, a high-mobility polymer channel semiconductor for high-performance OFETs has been fabricated via a facile solution process. The process capitalized on the cooperative role of polystyrene in assisting and facilitating the self-assembly of lower-MW, low-mobility (I) into an interpenetrating nanowire network semiconductor with significantly enhanced charge transport efficacy. When used as a channel semiconductor in solution-processed OFET devices, this nanowire network of (I) afforded field-effect mobility and current modulation in excess of 8 cm^2^ V^−1^s^−1^ and 10^7^, respectively. The enhancements are a factor of five better in mobility and over two orders of magnitude higher in current modulation than those of the corresponding semiconductor of lower-MW (I) without the assistance of polystyrene. These field-effect properties are essentially similar to those of very high MW, high-mobility (I), which are more than sufficient for many impactful electronic applications.

## Methods

### Materials

Lower-MW DPP-DTT polymer was synthesized according to previously reported procedure^10^ except that the polymerization was carried out for about 55 hours. The crude polymer product was purified by sequential Soxhlet extractions with ethanol, ethyl acetate and then chlorobenzene. The polymer obtained from the chlorobenzene extraction, which showed MW properties of *Mn* ~55 kg mol^−1^ and *Mw* ~199 kg mol^−1^ relative to styrene standards (GPC analysis at 160 °C using trichlorobenzene as eluent), was used for OFET fabrication. Monodispersed polystyrenes with *Mw* of 2.2 kg mol^−1^ (*Mw/Mn:* 1.01), 19.7 kg mol^−1^ (*Mw/Mn*: 1.03), 97.1 kg mol^−1^ (*Mw/Mn:* 1.03), and 301.6 kg mol^−1^ (*Mw/Mn*: 1.04), 1,2-dichlorobenzene, octadecyltrichlorosilane (OTS-18) were purchased from Alfa-Aesar and Sigma-Aldrich and used as received.

### Film deposition and characterization

The polymer films for characterization and for OFET fabrication were prepared as follows. The surface of a heavily doped p-Si wafer substrate with a 350-nm thick thermally grown SiO_2_ surface layer was first modified with OTS-18 according to previously published procedure^27^ except that OTS-18 was utilized instead of octyltrichlorosilane. The solutions of (I) and polystyrene were prepared by dissolution of (I) and polystyrene in 1,2-dichlorobenzene at a concentration of 4.0 mg/ml. The solutions for the (I)/polystyrene blends of various weight percentages (wt %) were then prepared by combining the solutions of (I) and polystyrene in appropriate volume ratios. The resulting (I)/polystyrene solutions were stirred at 60 °C for 12 h before use. The solutions were spin coated on OTS-18-modified silicon wafer substrates at 1500 rpm for 150 s under ambient conditions. The resulting films were left to dry in a glove box overnight at room temperature before characterization. UV-vis absorption spectra of the films were recorded on a HP 8453 UV-vis spectrophotometer. AFM (Veeco Digital instruments) and SEM (LEO 1530 Field Emission) instruments were used to characterize the morphology and topography of the films. AFM measurement was performed in the tapping mode under ambient conditions. Element distributions in the films were analyzed by XPS depth profile (PHI Model 5802). Out-of-plane GIXRD data were recorded using a Bruker D8 Advance system (Cu X-ray source, 45 kV and 30 mA). Grazing incidence angle was fixed at 0.2° and detector was scanned from 2° to 30°. Crystallinity and elementary analysis of nanowires of (I) were carried out using TEM (TecnaiG2 20 S-TWIN integrated with EDX).

### OFET fabrication and characterization

Bottom-gate, top-contact OFETs were fabricated using the polymer semiconductor films previously deposited on the OTS-18-modified silicon wafer substrates. Gold source/drain electrodes were deposited on the semiconductor film through a shadow mask with channel length (*L*) and width (*W*) features of respectively 80 or 120 μm and 1500 μm by thermal evaporation in vacuum chamber. The resulting OFET devices were electrically characterized, and optionally annealed at 200 °C for 5 min before subjecting to another electrical characterization. The field-effect characteristics of OFET devices were measured using a Keithley 2636B two channel source/meter in ambient environment at room temperature. The field-effect mobility in the saturation regime was calculated by using the equation: I_DS_ = μC_i_(V_G_–V_th_)^2^ (*W*/2*L*), where I_DS_ is the drain current, μ is the field-effect mobility, C_i_ is the capacitance per unit area of the gate dielectric layer, and V_G_ is the gate voltage. The field-effect mobility values were extracted from the |I_DS_|^1/2^ vs V_G_ plot in the low gate voltage (V_G_) regime (V_G_−V_th_ ~10 V)^40^.

## Additional Information

**How to cite this article**: Lei, Y. *et al*. Solution-Processed Donor-Acceptor Polymer Nanowire Network Semiconductors for High-Performance Field-Effect Transistors. *Sci. Rep.* **6**, 24476; doi: 10.1038/srep24476 (2016).

## Supplementary Material

Supplementary Information

## Figures and Tables

**Table 1:** Field-effect properties of (I)/polystyrene semiconductors in OFETs.

**(I)/polystyrene (wt%)**	**Mobility (c**m^2^ **V**^−**1**^**s**^−**1**^)		**On/off ratio**	**V**_**th**_ **(V)**
	**Max. value**	**Ave. value**		
100/0	1.51	1.16	2 × 10^5^	9.7
80/20	4.05	3.39	2 × 10^6^	6.4
60/40	6.98	5.80	3 × 10^6^	6.3
40/60	**8.25**	**6.76**	**1 **×** 10**^**7**^	**1.7**
20/80	3.34	2.57	1 × 10^7^	4.6
