# Supplementary material for: Solution-Processed Donor-Acceptor Polymer Nanowire Network Semiconductors For High-Performance Field-Effect Transistors
Source: Sci Rep. 2016 Apr 19;6:24476. doi: 10.1038/srep24476 (PMC4835732; doi:10.1038/srep24476)
Supplement: Supplementary Information [file srep24476-s1.pdf]

Supplementary Information

Solution-Processed Donor-Acceptor Polymer Nanowire Network Semiconductors for High-Performance Field-Effect Transistors

Yanlian Lei[1], Ping Deng[2], Jun Li[3], Ming Lin[3], Furong Zhu[1], Tsz-Wai Ng[4], Chun-Sing Lee[4], & Beng S. Ong[2,5]

[1] Department of Physics and Institute of Advanced Materials, Hong Kong Baptist University, Hong Kong SAR, P. R. China.

[2] Research Centre of Excellence, Institute of Creativity and Department of Chemistry, Hong Kong Baptist University, Hong Kong SAR, P. R. China.

[3] Institute of Materials Research and Engineering, Agency for Science, Technology and Research, Singapore 117602

[4] Center of Super-Diamond and Advanced Films, Department of Physics and Materials Science, City University of Hong Kong, Hong Kong SAR, P. R. China.

[5] Department of Materials Science and Engineering, South University of Science and Technology of China, Shenzhen, Guangdong, P.R. China.

Correspondence and requests for materials should be addressed to B.S.O. (email: bong@hkbu.edu.hk) or to F.R.Z. (email: frzhu@hkbu.edu.hk)

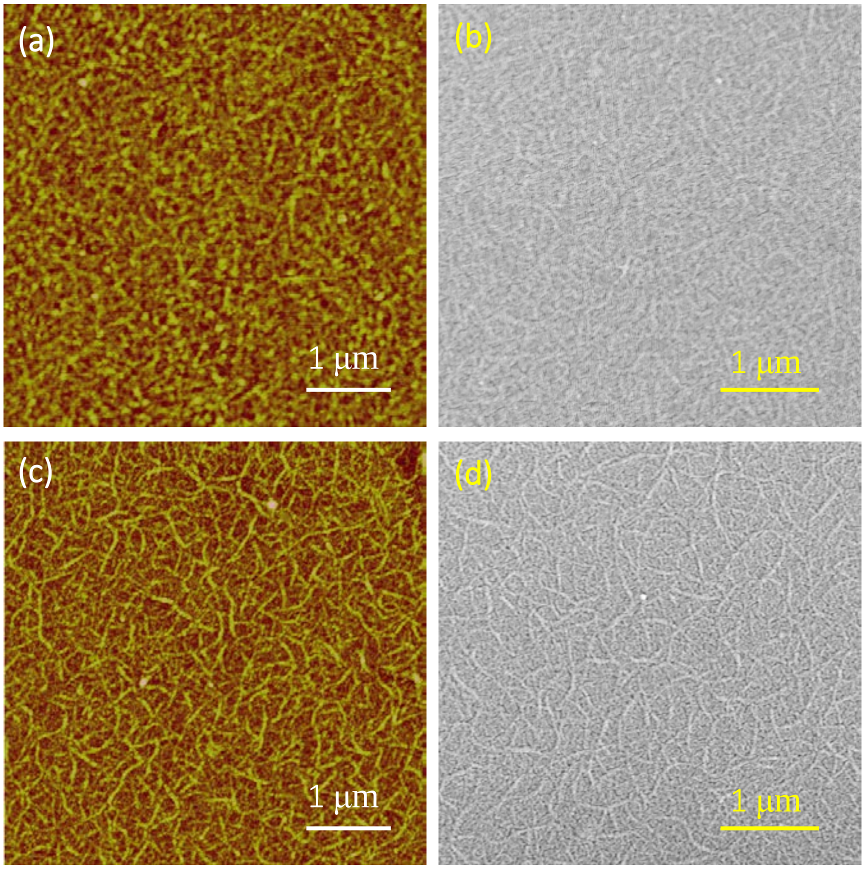

Figure S1. AFM and SEM images of nanowire networks of (I) from (I)/polystyrene (40/60 wt %) films. (a) and (b) are AFM (topography) and SEM images of defused nanowire-like features of (I) from (I)/polystyrene film thermally annealed after polystyrene removal; (c) and (d) are AFM (topography, same as Figure 2f) and SEM images of well-defined nanowire network of (I) from thermally annealed (I)/polystyrene film with the polystyrene removed after thermal treatment.

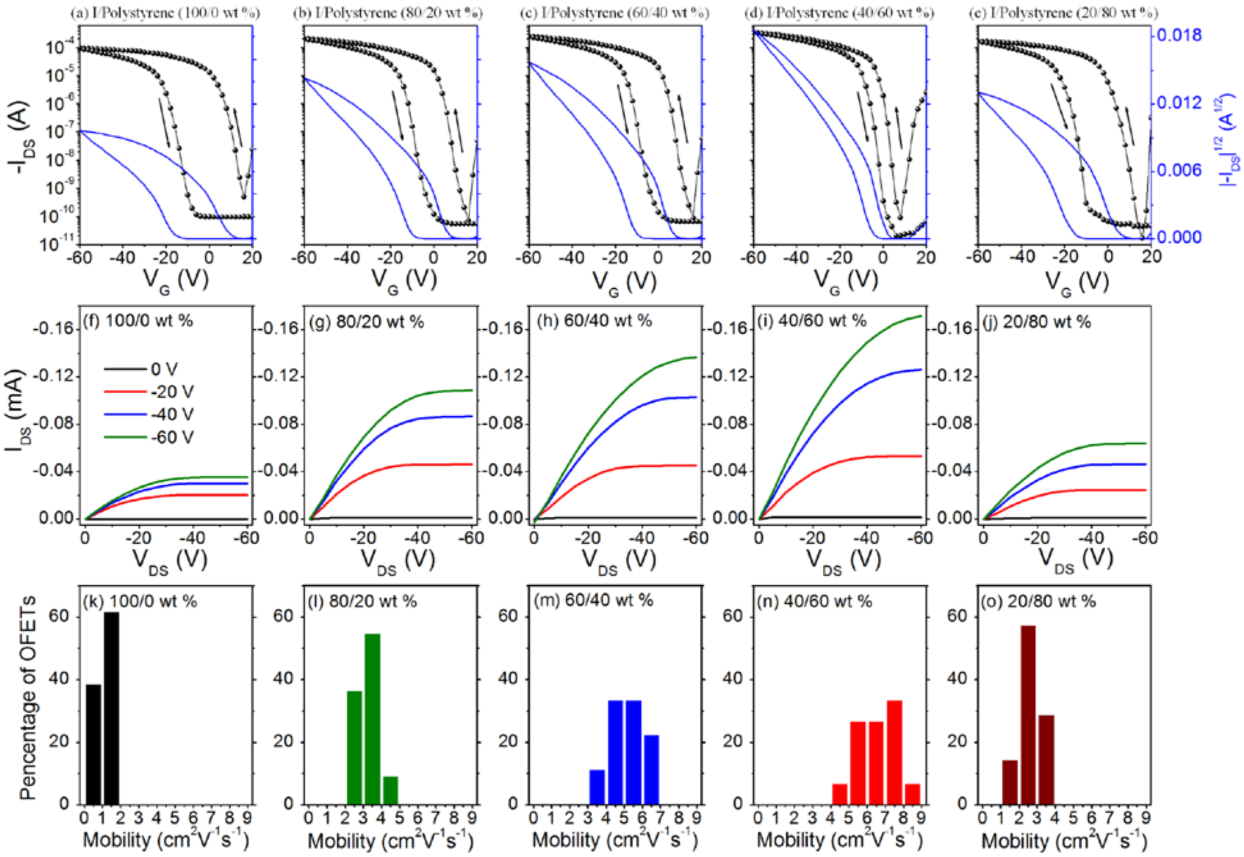

Figure S2. Field-effect properties of OFETs with (I) and (I)/polystyrene channel semiconductors. Typical transfer characteristics of OFETs with (a) neat (I), and (b)-(e) (I)/polystyrene channel semiconductors with various polystyrene loadings; typical output curves of OFETs with (f) neat (I), and (g)-(j) (I)/polystyrene channel semiconductors with various polystyrene loadings; Mobility distributions of OFETs with (k) neat (I), and (l)-(o) (I)/polystyrene channel semiconductors with various polystyrene loadings.

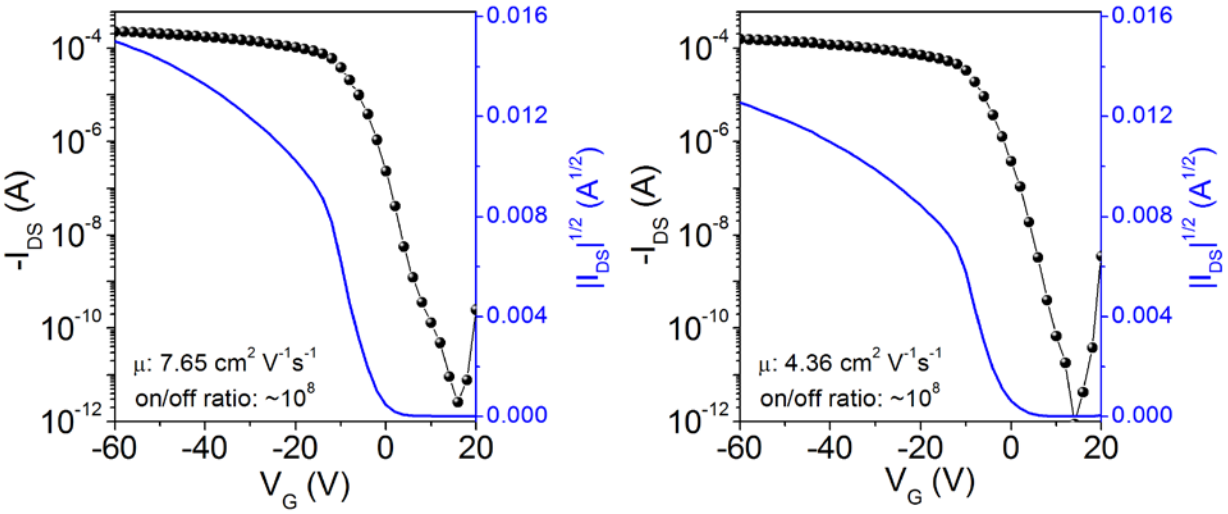

Figure S3. Field-effect transistor performances based on annealed films with polystyrene removal. (a) Annealed with polystyrene; (b) annealed without polystyrene.
